# Main Complications of Mild Induced Hypothermia after Cardiac Arrest: A Review Article

**DOI:** 10.5681/jcvtr.2014.001

**Published:** 2014-03-21

**Authors:** Hassan Soleimanpour, Farzad Rahmani, Samad EJ Golzari, Saeid Safari

**Affiliations:** ^1^Cardiovascular Research Center, Tabriz University of Medical Sciences, Tabriz, Iran; ^2^Emergency Medicine Department, Tabriz University of Medical Sciences, Tabriz, Iran; ^3^Liver and Gastrointestinal Disease Research Center, Tabriz University of Medical Sciences, Tabriz, Iran; ^4^Anesthesiology and Critical Care Department, Iran University of Medical Sciences, Tehran, Iran

**Keywords:** Cardiac Arrest, Hypothermia, Cardiopulmonary Resuscitation

## Abstract

The aim of the present study is to assess the complications of mild induced hypothermia (MIH) in patients with cardiac arrest. Presently, based on the guidelines of the American heart Association, MIH following successful cardiopulmonary resuscitation (CPR) in unconscious adult patients due to ventricular fibrillation (VF) with out-of-hospital cardiac arrest (OOHCA) is essential and required. However, MIH could be associated with complications in Patients with cardiac arrest. Studies conducted on the precautions and care following cardiac arrest and MIH were included. Valid scientific data bases were used for data collection. The obtained results from different studies revealed that mild MIH could be associated with numerous complications and the knowledge and awareness of the medical staff from the complications is required to guarantee successful therapeutic approaches in MIH following cardiac arrest which is a novel medical facility with different styles and complications. Overall, further future studies are required to improve the quality of MIH, to increase survival and to decrease complications rates.

## Introduction

Resuscitation has been described for the first time by Vesalius almost 500 years ago. However, modern CPR emerged almost years ago.^1,2^ Moderate hypothermia induction following cardiac arrest was firstly described in 1950; however, it was abandoned without further investigations.^3-5^ MIH regained popularity for protection of the patients from neurological damage in the 1980s. MIH was then used for reducing cerebral metabolism in some cardiac and brain surgeries. Recently, MIH is under attention in the witnessed cardiac arrest and successful resuscitation. The main reason for the increased prevalence of MIH in patients with cardiac arrest is the protective mechanisms against cerebral hypoxic damages and the associated with good neurological outcomes.^6,7^ MIH as also been used in patients with brain traumatic injury for controlling refractory increased intracranial hypertension.^8-12^ In November 2005, the advanced life support task force of the international liaison committee on resuscitation suggested MIH in adult patients with impaired consciousness following return of spontaneous circulation (ROSC) after OOHCA with primary rhythm of ventricular fibrillation.^4,13-16^ Furthermore, this committee have emphasized on the requirements of further investigations on patients with other cardiac arrest cases such as in hospital cardiac arrest.^17^

MIH, despite numerous advantages, if associated with some complications, the normal function of most enzymes is temperature-dependent. Therefore, most enzymatic reactions such as metabolism of medications are impaired. Furthermore, other physiological activities including blood circulation, respiration, coagulation and hepatic and renal activities are impaired following MIH.^18,19^ Although most changes following MIH are physiological, these changes are not appropriate for the critically ill patients. Some of the complications associated with MIH such as bradycardia will not require any specific treatment. However, other complications such as hyperglycemia due to the associated increased risk of infection and negative effects on neurological functions require serious treatments.^6,18-20^ Considering the serious complications of MIH, immense attention should be paid on the indications and inclusion criteria of the patients for MIH.^21^ Complications could be observed at every stage of MIH (induction, maintenance and rewarming) and should be monitored closely to increase the survival rate and decrease mortality rate and other complications.^18-19^

## Methodology

Articles used in this review were accessed from the available evidence on the Hypothermia after Cardiac Arrest. The following keywords were used: Post-CPR management; Hypothermia after Cardiac Arrest; Complication of Mild Induced Hypothermia. Firstly, we searched for systematic reviews, evidence-based clinical practice guidelines, health technology assessments, and randomized controlled trials. In addition, in order to achieve a better conclusion, we used the following data bases and sites:

1. Cochrane library

2. PubMed

In this manuscript we just reviewed the published articles, books and guidelines from 1959 up to 2013 and our criteria for inclusions and exclusions were as follows:

### Inclusion Criteria

- Studies on Hypothermia after Cardiac Arrest (HACA)
- Studies on CPR and post CPR care
- Studies on complication of hypothermia
- Studies performed in adult age group

### Exclusion Criteria

- Studies published in a language other than English and Persian

## Analysis

**Figure 1:**
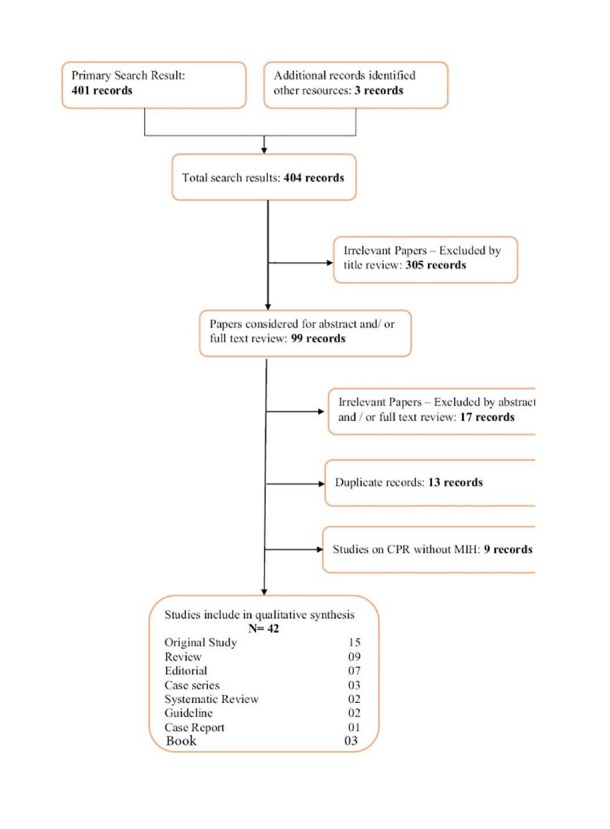
Flow Chart of Study

The search strategy resulted in 404 articles, books and guidelines. The irrelevant papers by title review (305) were excluded leaving 99 articles. Forty two articles, books and guidelines were selected for further analysis including 15 original studies, 9 review articles, 7 editorial, 3 case series, 3 books, 2 systematic reviews, 2 guidelines, 1 case report meeting our criteria (Figure 1). The remaining 57 articles were excluded due to the following reasons: Irrelevant abstracts or full-text review (17 articles), duplicate records (31 articles) and studies on CPR without mild induced hypothermia after cardiac arrest (9 articles). The characteristics of all the included studies are shown in Table 1.

**Table 1:** Characteristics of all the included studies in our review

**Year**	**Authors**	**Study Design**	**Subject of Study**
1959	Safar et al.	Review	Upper airway obstruction in unconscious patient
1960	Kouwenhoven et al.	Original	CPR
1959	Benson et al.	Review	Review of MIH
2004	Bernard et al.	Original	Therapeutic Hypothermia
2002	Holzer et al.	Original	Neuroprotective effect of MIH
2007	Milanovic et al	Review	Adverse Effect of Hypothermia
2005	Nolan et al.	Guideline	Resuscitation Guideline
2004	Tisherman et al.	Review	Adverse Effect of Hypothermia
2011	Moore et al.	Review	Therapeutic Hypothermia
2007	Brain Trauma Foundation	Guideline	Traumatic Brain Injury Guideline
2008	Peterson et al.	Systematic Review	Hypothermia treatment for traumatic brain injury
2011	Tuma et al.	Case Series	Use of Hypothermia for Traumatic Patients
2005	Hazinski et al	Editorial	Controversy in Resuscitation
2005	Nolan et al.	Editorial	Controversy in Resuscitation
2003	Nolan et al.	Editorial	Therapeutic Hypothermia
2002	Safar et al.	Review	Therapeutic Hypothermia
2006	Alzaga et al.	Review	Therapeutic Hypothermia
2006	Behringer et al.	Book	Therapeutic Hypothermia
2013	Soleimanpour et al.	Book	Therapeutic Hypothermia
2006	Luscombe et al.	Review	Therapeutic Hypothermia
2006	Kabon et al.	Review article	Therapeutic Hypothermia
2001	Zheng et al.	Original	Epidemiology of Cardiac Arrest
2011	Bessen et al.	Book	Hypothermia
2002	Bernard et al.	Original	Therapeutic Hypothermia
2001	Ujhelyi et al.	Original	Defibrillation Therapy in Hypothermia
2006	Storm et al.	Editorial	Therapeutic Hypothermia
2004	Kochanek et al.	Editorial	Therapeutic Hypothermia
2007	Cushman et al.	Original	Therapeutic Hypothermia
2003	Nolan et al.	Editorial	Therapeutic Hypothermia
2011	Testori et al.	Original	Therapeutic Hypothermia
2007	Manoukian et al.	Original	Outcome of Acute Coronary Syndrome
2009	Schefold et al.	Original	Coagulopathy due to Hypothermia
2012	Soleimanpour et al.	Editorial	Capnography in Emergency Department
2013	Soleimanpour et al.	Original	Capnography in Emergency Department
2011	Michelle et al.	Review	Therapeutic Hypothermia
2011	Haugk et al.	Original	Therapeutic Hypothermia
2012	Nielsen et al.	Systematic Review	Review of MIH after cardiac arrest
2012	Soleimanpour et al.	Case Series	Drugs
2012	Soleimanpour et al.	Original	Drugs
2003	Schwarz et al.	Original	Drugs
2010	Soleimanpour et al.	Case Report	Drugs
2011	Soleimanpour et al.	Case Series	Drugs
2012	Soleimanpour et al.	Original	Drugs

### Cardiovascular effects and hemodynamic parameters changes

The associated complications of MIH mostly affect cardiovascular systems. Considering the fact that almost 80% of the patients with cardiac arrest at the background of cardiac diseases and almost 60% of the mortality cases in adults are due to coronary artery disease, hypothermia could deteriorate cardiac dysfunction and hemodynamic imbalance.^7,22^ MIH could increase catecholamines into circulation and consequently increase cardiac output and myocardial oxygen demand.^20^

MIH (32-34 °C) caused significant changes in the hemodynamic parameters leading to loss of cardiac contractility and decreased heart rate (cardiac output decreased by 25%). In addition, central venous pressure and arterial resistance increase and a slight increase occur in blood pressure (about 10 mmHg) due to vasoconstriction. The possibility of the occurrence of such complications in the cerebrovascular system (brain vessels) is low. This process creates a balance between cerebral blood flow and its metabolism (based on the measurement of glucose and oxygen consumption) and may even improve it. It is worth mentioning that such effects in different studies on adults and children have been confirmed.^19,23^

Hypothermia may also cause changes in the ECG and cardiac rhythm. At the time of induction of hypothermia and a drop in body temperature, shift of circulating blood volume from the peripheral circulation to the central circulation and also an increase in venous return cause a sinus tachycardia. When the body temperature falls below 35.5 °C, a sinus bradycardia occurs and this situation is exacerbated by the temperature drop. (For example, at a temperature of about 32 °C, the heart rate is about 40 beats per minute or less).^6,19,20^ In comparison with normothermic group, Patients undergone hypothermia induction have lower cardiac index and higher peripheral vascular resistance.^24^ Decrease in heart rate is due to the decrease in diastolic depolarization of sinoatrial node cells and ECG changes, including increasing the intervals or distance between different waves (P-R, Q-T) and also widening of the wave of ventricular depolarization (QRS) and sometimes the presence of Osborn wave.^20 , 23^

As noted earlier, diastolic and systolic dysfunction cause decreased contractility of myocardium, leading to a 25% decrease in cardiac output. In general, the lower metabolic rate during hypothermia is approximately equal to or even greater than the reduction in cardiac output. Central venous blood oxygen saturation (SvcO_2)_ remains unchanged or may increase due to stability or improvement in blood flow. Hypothermia-induced bradycardia usually does not require treatment. However, if treatment is necessary, atropine will not be effective because of the mechanism of hypothermia-induced bradycardia and other treatments, such as isoprenaline, slight warming-up the patients or rarely; and in very severe cases, transvenous pacing or permanent pacemaker should be applied.^19,23^ As mentioned previously, cardiac arrest mostly occurs in the background of myocardial ischemia. Therefore, ischemia is able to worsen the potential arrythmogenic effects of hypothermia.^25^

The incidence of serious arrhythmias, when the temperature is above 30 °C, is very low. However, when core body temperature reaches about 28-30 °C; the incidence of life-threatening arrhythmias increases, especially if there are concomitant electrolyte abnormalities. Arrhythmia usually starts with atrial fibrillation and ultimately turns into ventricular tachycardia or ventricular fibrillation. Hypothermic myocardium is very sensitive to any mechanical manipulation and any indiscretion will lead to the conversion of cardiac rhythm from atrial fibrillation to the ventricular fibrillation. It should be considered that hypothermic myocardium is resistant to anti-arrhythmic drugs. Therefore, care must be taken to keep the temperature above this range.^15,18^

### Ischemia and coronary blood flow

According to published studies, hypothermia leads to an increased risk of myocardial infarction due to resulted coronary vasoconstriction. Myocardial ischemia in hypothermic patients depends on the previous status of coronary artery in patient, so that in normal individuals, hypothermia has been shown to improve myocardial blood flow; however, in patients with a history of coronary artery disease, it causes vasoconstriction in atherosclerotic arteries.^18^ Following ROSC in patients with VF, myocardium is mechanically and electrically unstable. This would contribute to restart of fibrillation instantaneously following ROSC. Recurrence of VF would be dangerous for the patients if MIH is initiated soon after ROSC.^15,24^

Different studies on animals and also review of initial studies shows that if hypothermia is started in the early stages of treatment, it may reduce cardiac damage caused by cardiac arrest.^19,26^

### Electrolyte abnormalities

In hypothermic patients, serum electrolyte disturbance occurs because of the increased renal excretion of electrolytes and the resulted intracellular shift. The reason for the increased renal excretion includes changes in volume adjustment in blood circulation, cardiac preload and also impaired tubular function. Electrolyte abnormalities, particularly magnesium are very important, because they are associated with neurological adverse consequences. Magnesium deficiency in patients with brain trauma may cause adverse neurological outcome and administration of magnesium may reduce secondary damages and death of cells in the cerebral cortex. Another important role of magnesium is to prevent the damage caused by reperfusion. In addition, magnesium deficiency is associated with brain and coronary vasoconstriction. Several studies have shown that administration of magnesium after myocardial infarction is associated with reduced infarct size and improvement of remained myocardial function. Also, magnesium deficiency is associated with atrial and ventricular arrhythmia, bronchospasm, seizures and metabolic effects such as insulin resistance. Magnesium deficiency can also lead to other electrolyte abnormalities such as hypokalemia, hypocalcemia, hyponatremia, hypophosphatemia.^18,27,28^

Clinical trials indicated that magnesium deficiency is an independent predictor for the adverse outcomes both in critically ill patients admitted to the intensive care unit and in patients admitted in general ward units. Magnesium deficiency is particularly associated with complications in patients with unstable angina or myocardial infarction. Magnesium administration reduces mortality, reduced size of infarction area by vasodilation coronary arteries, antiplatelet activity, and automaticity suppression. It also protects cardiac myocytes against the entry of calcium into cell during reperfusion phase in such patients.^19^

Hypokalemia and hypophosphatemia can also have adverse effects, such as arrhythmia, muscle weakness and neuromuscular disorders. Hypophosphatemia causes weakness of the diaphragm muscle and respiratory muscles, increased risk of respiratory infections and delay in weaning the patient from the mechanical ventilator. Hypophosphatemia in children causes myocardial dysfunction and reduced cardiac output.^18^

Clinical effects of hypokalemia include cardiac arrhythmias, muscle weakness, rhabdomyolysis, renal failure, and elevated levels of blood sugar (due to suppression of insulin secretion). Risks of sodium disturbance in the neural injury are unknown. Both hyponatremia and hypernatremia may exacerbate brain injury. Potential complications of electrolyte disturbance indicate that preventing electrolyte imbalance caused by hypothermia should be the main target of treatment in hypothermic patients.^18,29^

Blood levels of magnesium, potassium, and phosphorus in patients with neural injury should be kept in normal or above normal status and we should also note that the serum level of magnesium does not always represent the actual amount of magnesium in the body, because the amount of intracellular magnesium may be extremely low, while serum magnesium levels are normal. In this situation, the ionized magnesium level will be a better indicator for the active magnesium level of the body.^19^

### Hyperglycemia

Insulin reticence and decreased in insulin release would contribute to hyperglycemia in patients with MIH. In critically ill patients, hyperglycemia has been reported to be associated with high morbidity and mortality.^21^ As previously described, hypothermia decreases insulin sensitivity and also decreases insulin secretion by the pancreas. Therefore, patients treated with hypothermia are exposed to the risk of hyperglycemia; and increased blood sugar levels are associated with increased morbidity and mortality. Tight control of blood sugar levels and insulin therapy has been associated with decreased morbidity and mortality. Increased blood sugar levels are associated with increase in the rate of infection, neuropathy and renal failure. Therefore, in hypothermic patients, tight control of blood sugar levels is essential.^19,30^

### Other metabolic effects and blood gas levels

Hypothermia increases the production of glycerol, free fatty acids, ketones and lactate leading to mild metabolic acidosis, without the need for special treatment. Usually extracellular hydrogen concentration is measured, but there is a slight increase in the level of intracellular hydrogen during hypothermia. Reduction in metabolic rate (5-8% per one degree decrease in core body temperature) decreases oxygen consumption and carbon dioxide production. Therefore, Mechanical Ventilation Settings should be adjusted during hypothermia and blood gas levels should be checked periodically.^6,19,20^ Since blood gas values are affected by blood temperature and the blood gas analyzers bring the blood temperature to 37 °C and then the analysis is conducted, the arterial partial pressure of oxygen and arterial partial pressure of carbon dioxide increase (due to increased solubility of gases following increase in temperature) and concentration of blood hydrogen ion also increases falsely.^18,23^

### Coagulation system

MIH causes a mild increase in an individual’s susceptibility to bleeding. This increase is realized through the effect on the number and functions of platelets, production of clotting enzymes, tissue plasminogen activator inhibitor enzyme, other steps in the coagulation cascade and also increased bleeding time.^6,19^ Throughout hypothermia, platelets become sequestrated in the spleen and liver and reenter the circulation after rewarming.^20^ Coagulopathies associated with hypothermia would question the safety of the procedure in patients following revascularization of the coronary arteries with fibrinolytic medications or percutaneous coronary intervention (PCI).^31^ However, MIH in patients following reperfusion procedures have not been associated with increased bleeding risk.^32^ Similar to the analysis of blood gases, standard tests of blood coagulation are reported to be normal due to temperature rise in blood temperature in the device.^20,23^

### Infection

MIH impairs immune function and inhibits many inflammatory reactions. Its anti-inflammatory effects can act as a protective factor against brain injury. Hypothermia inhibits the secretion of cytokines and suppresses the migration of leukocytes and phagocytes. Insulin resistance and hypothermia–induced hyperglycemia also increase the risk of infections.^6,18,19^ The infection incidence is higher in hypothermic compared to normothermic patients (19% versus 6%, respectively).^6^ Nosocomial pneumonia was observed in more than 50% of the patients having undergone hypothermia induction for more than seven days.^20^ But if hypothermia persists for 24 hours or less, increased risk of infection will be minor or not exist at all. Decreased motility of the intestine in patients having undergone hypothermia would contribute to the increased risk of infection. Therefore, it is required for patients having undergone hypothermia used prokinietic medications improved intestinal motility. In some centers, selective decontamination of gastrointestinal tract is used for decreasing infection rates.^20^ HACA researchers reported sepsis as the most important complication of MIH; their report, however, was not statistically significant.^5^

There is an increased risk for bed sores infections due to the effects of hypothermia on cutaneous vasoconstriction and reduced performance of leukocytes, and thus the meticulous care of these patients is recommended to prevent bed sores (because the condition of sores often worsens and wound healing is disrupted). The last point is that the areas of vascular catheters and other surgical wounds should be considered.^18^

### Respiratory system

In patients with MIH in order to prevent respiratory alkalosis and hypocapnia, it is required to set the ventilatory volumes in the least possible amounts due to the low metabolism rate and decreased carbon dioxide production.^20,33,34^

### Renal system

Cold diuresis is a major concern in patients with MIH. It occurs due to decreased reabsorption of the solutes in the ascending Loop of Henle. To maintain proper intravascular volume is of great importance in these patients in order to prevent hypotension. Diuresis would be associated with fluid and electrolyte imbalance in these patients.^21^ Throughout rewarming, electrolytes would shift to the extra-cellular compartment and increase plasma concentrations.^18,20^

The relative increase in venous return activates the secretion of atrial natriuretic peptide (ANP) and reduces antidiuretic hormone levels (ADH). This situation in combination with other mechanisms such as tubular dysfunction increases the urine output which is known as “cold diuresis.” If diuresis is not untreated, then hypovolemia, renal excretion of electrolytes, hemoconcentration, and increased blood viscosity will be caused. Risk of reduced volume, particularly when patients are treated with diuresis enhancing factors (mannitol on traumatic brain injury) increases. Increased blood viscosity (2% for every degree of core temperature decrease) causes obstruction of blood flow in microvascular system.^19^

Described mechanisms along with renal tubular dysfunction cause significant electrolyte disturbance and elevated levels of sodium and serum osmolality. Therefore, one must pay due attention to the intravascular volume and fluid balance in a hypothermic patient and occurrence of hypovolemia should be avoided, and in case of occurrence, it should be treated promptly.^18^

### Shivering

In awake patients, MIH could be associated with shivering which increases oxygen consumption.^21^ At the time of induction of hypothermia, body uses several mechanisms to obtain and maintain the heat. At this status, the body prevents lose of temperature through increasing sympathetic tone and vasoconstriction of skin and uses shivering to generate heat. Shivering increases oxygen consumption about 40 to 100% and has adverse effects on patients with neurological injuries and damages caused by hypoxia. This is less of a problem in patients undergoing mechanical ventilation, because shivering does not lead to increased respiratory effort in these people.^19,35^

In most cases, shivering is controlled with a low dose of narcotics. In cases where the use of muscle relaxants or drugs is improper, alternative therapies such as the use of neostigmine, clonidine and ketanserin is recommended, but side effects of these drugs should always be taken into consideration (For example, clonidine can exacerbate hypothermia-induced bradycardia).^19,36^

### Drug metabolism

Hypothermia is clearly effective on drug metabolism and pharmacokinetics (due to effects of temperature dependence of enzyme). Effects of temperature on the most drugs metabolism and clearance used in the intensive care unit or the emergency room are unknown, but it seems that the clearance of most drugs (Propofol, muscle relaxants, fentanyl and barbiturates, etc.) is reduced by hypothermia.^18,37,38^ The efficacy of most medications used in CPR could decrease or their effects could appear with delay. Lidocaine has no confirmed effects throughout hypothermia. Also, amiodarone is not useful in controlling fibrillation in the hypothermic heart.^7,39-42^

### Other complications

Hypothermia increases serum levels of amylase and liver enzymes.^6^ In addition, most patients undergoing hypothermia induction would require intubation, mechanical ventilation, sedation and muscle relaxation. These would limit evaluation and monitoring of neurological function of the patients.^21^ Fortunately, although hypothermia has adverse effects, but most of the complication of induction of hypothermia can be prevented or controlled in intensive care conditions. Problems related to patient handling are different depending on the depth and duration of hypothermia and also their underlying disease. Generally, the use of hypothermia in patients with traumatic brain injury and stroke causes more problems than the use of hypothermia in patients after cardiac arrest and resuscitation. Finally, it is important that all medical and nursing staff be aware of the physiological and pathophysiologic changes that can occur due to hypothermia and they must know which side effects require treatment and which ones do not require any treatment.^18^

## Conclusion

The present study provides information on the complications of MIH in patients following cardiac arrest. Considering the previously conducted studies and the present study, it is essential to have a comprehensive knowledge of the complications associated with MIH in patients with cardiac arrest and the medical/nursing staff working in the emergency department and Intensive Care Unit (ICU) should be aware of the complications. Furthermore, the researchers working in the field of HACA to conduct studies on the associated complications of this novel approach so that the required preventive measures are found and patients with cardiac arrest would benefit the increased survival rate following administration of MIH.

## Ethical issues

Not applicable.

## Competing interests

The authors declare that they have no conflict of interest.
